# A hopeful tale from the wonderland of psychosis

**DOI:** 10.1038/npjschz.2015.15

**Published:** 2015-08-26

**Authors:** Berenice Royal

I am Berenice Royal. I have paranoid schizophrenia. Berenice is not my real name; I use a pseudonym because I don’t want the stigma connected with my illness to impact negatively on my life more than it already does. I have managed to avoid many of the negative consequences that others with schizophrenia face. I hold a PhD in one of the Social Sciences and work as a scientist at a UK university. I love my work, and I have published several books in my field and received lots of great feedback on my teaching.

In this essay, I want to tell you how my illness developed, how I experience my illness and explain it to myself, and what I do to help myself. I hope my experiences will help others to overcome the limitations of schizophrenia and succeed in life.

## The evolution of an illness

I have to start with my childhood. There is some evidence that childhood trauma can cause schizophrenia.^1^ While my parents were extraordinarily loving and caring, I experienced some trauma nonetheless. I was born in East Germany in 1976 to parents who were critical of the regime. I avoided being recruited into the system at a very young age: I had good grades and was offered access to an elite school, but refused because it would lead into a career in the state system and separate me from my family. Later, teachers warned my friends’ parents that I would be a bad influence on their children. My family was observed by the Stasi, which was scary, but we also made fun of them to relieve the anxiety. We were finally allowed to leave the GDR nearly one year before the wall came down on 5 November 1988. It will always be a memorable day for me. We were given 48 h to leave, which made the whole process traumatic, and I believe that my experiences in the GDR relate to some of the symptoms I later developed.

When we arrived in West Germany, life was a struggle because we did not have a lot of money. Nonetheless, I excelled at school. When I entered university, I fell deeply in love with a friend who also studied one of the social sciences. Heartbroken, as he did not seem to return my feelings, I decided to move back to Berlin, which in the meantime had been unified. That decision was probably crucial for my further life. I was quite isolated in the big city, and isolation and urban living can contribute to the development of schizophrenia.^2,3^ However, I had a great job at the University, which I loved, and for a while things went well.

Two years later 9/11 happened. Like everyone else, I was shocked. No one had predicted or could really explain the attacks. The attacks had a huge effect on me: I became obsessed with reading speculations about the causes and even got into conspiracy theories. At the same time, I got back in contact with my friend, who had also moved to Berlin. We decided to meet, and I thought I might finally find the courage to tell him about my feelings. However, two days before we were due to meet he was killed in a car crash. In the same year, I lost my job at the University. I believe that all of these events together contributed to the outbreak of my illness. I had a series of what felt like revelations over a couple of days, which I have described elsewhere,^4^ and following that became psychotic and paranoid. Finally, my parents intervened and got me to hospital, where I was diagnosed.

I decided to take the medication I was prescribed, but still to continue with my life. I took up a prestigious fellowship in the US. I loved my time there, and when I came back I applied for a job as a teaching fellow at a university and started a PhD. During this time, my symptoms were reasonably well controlled as long as I took my medication. I did suffer from some depression and hypochondria, but these could be considered normal after all that I had experienced and could also be described simply as grief and fear of further illness. During my first job after graduation, I fell in love with a man from the UK. It was a difficult but stimulating relationship; we argued a lot about current affairs, and he inspired me to write a book. He lived with me for a while in Germany; when he moved back to England I decided to follow him. However, as soon as I had moved to England, the relationship ended.

That was the beginning of the second period of my illness. I started to have more serious symptoms again. Nevertheless, I decided to stay in England and make a success of my job. I finished my PhD, published six books and loved teaching more the better I got at it. By now, seven years later, I really love everything about where I am in my life and I am just starting a relationship with a lovely man who is a journalist.

## My symptoms of schizophrenia

Let me tell you a bit about my symptoms. Sometimes, I have pleasant symptoms. My first episode of revelations was entirely positive and very spiritual. After that, I believed myself to be in telepathic contact with one of my teachers, who was travelling to Russia, and I started to hitchhike across Germany in pursuit of him. I often have the perception of being telepathically connected to men, which is usually much less pleasant. Among my other experiences, I also have seen and heard ghosts, and I had an LSD-like experience of seeing a fish goddess and being led through some spiritual realms.

Sometimes, I simply have paranoia. Once I believed I was Edward Snowden; at other times I fear persecution or punishment for some unknown sin. I often believe that I am being observed by the intelligence services, which I think relates to my past experience and the current media discussions about surveillance. My hallucinations can sometimes be destructive: Once I had physical hallucinations of violence against me. That was nearly as painful as real violence would have been. On the other hand, I sometimes have helpful symptoms, such as voices that give me practical advice.

My ‘negative’ symptoms include not wanting, or being able, to talk much, which I think is related to my positive symptoms. In some ways I experience a different reality, which is difficult to share with other people.

I view my symptoms as being related to trauma and my subconscious; they often feel like telepathy and similar spiritual experiences.^5^ Traumatic experiences, such as 9/11, have resulted in this illness in many people (cf. refs 6, 7). In addition, poverty and migration, which can be associated with traumatic experiences, are risk factors for schizophrenia. Schizophrenia could be caused by the patient internalizing traumatic memories and thinking about them too much, which can be self-destructive. It is known that patients with schizophrenia often have posttraumatic stress disorder.^8,9^ This might explain why some people with schizophrenia become aggressive at times (posttraumatic stress disorder and aggression are linked, cf. ref. 10). On the other hand, the ‘overthinking’ about traumatic events might explain the negative symptoms of schizophrenia (such as apathy, lack of motivation, not talking). There has already been some work on trauma as a cause of schizophrenia, as well as a book on overthinking and schizophrenia.^11^

This idea might also explain why patients are prone to smoking and drinking a lot of coffee (cf. refs 12, 13), as both stimulants can enhance cognitive ability or at least concentration, which is needed when thinking a lot. On the other hand, it is also possible that both substances in themselves cause or worsen schizophrenia. Tobacco was originally used for its hallucinogenic properties and caffeine is thought to contribute to anxiety in sensitive individuals, which could contribute to paranoia. Consistent with the idea that patients with schizophrenia think too much, they also show abnormal neurological activity, and this too could be explained by the ingestion of too much caffeine or nicotine. Also, both substances were introduced into Europe about 100 years before the alleged reported increase in schizophrenia in Europe.^14^ They were probably first consumed in the upper classes, where the initial increase took place, and only much later widely and in the lower classes.

## Living life with schizophrenia

Despite all these symptoms, people are sometimes amazed at how much I have achieved. I am quite competitive by nature, which helps. I want to share some of the tips and tricks that I learned over the years. Of course, the most important approach for many (not all though, cf. ref. 15) patients is taking some medication. I favor keeping medications to a minimum, as the side effects can be destructive. For example, I am taking 2–4 mg Risperidone along with Citralopram. If I take more than this, I become drowsy and sleep a lot; naturally this interferes with my normal life. I also find that maintaining my weight with a healthy diet (pescetarian, vegetarian or vegan) and controlling my intake of stimulants and alcohol are very helpful.^4^ Another approach that I use is orthomolecular medicine, which refers to the use of mega-doses of certain vitamins (Niacin, Omega 3, and Vitamin C) to treat mental illness. It is not widely accepted, but I believe that it shows great promise (cf. refs 16, 17). All the studies that have been done on it, to my knowledge, have been short term, but longer-term studies are essential, as proponents of this approach recommend that chronically ill patients follow the regimen for at least 5 years.

I also use what might be called ‘biblio-therapy’; I read a lot, both as part of my job as a scientist and in my own time, particularly on psychology and self-help books. Much of my reading has helped me to deal with my symptoms better. For example, *How to Win Friends and Influence People* by Dale Carnegie helped me a lot to overcome my problems in forming and maintaining friendships.^18^

I sometimes use mantras to control the voices. At times, I can change the content of the voices according to what I think.

There is still a lot of stigma around mental illnesses such as schizophrenia. In my experience, it helps to refer to my illness not in terms of my diagnosis, but rather simply as a neurological illness, which sounds less frightening. I also apply spiritual approaches relating to acceptance of others, compassion, and love to become a more likeable person, and this naturally helps with relationships. Nevertheless, isolation has been a big problem. I am lucky to have extremely loving and supportive parents and good friends and work colleagues who are tolerant and friendly and have helped me through tough times. My psychiatrist and many other people are also important in my life and have helped me greatly. I am also friendly and social by nature, and that has helped me overcome the stigma and isolation a little bit. My social side does not always come through, though, when I have symptoms. Paranoia is the antithesis to sociability!

In addition, I use writing as a therapy. When I don’t write academically, I write in my journal. Writing has helped me to overcome loneliness and to solve many problems. I am a habitual internet and facebook user, which helps me to get useful information and to meet a lot of people.

I hope that people with schizophrenia or their carers will find useful information in my account. I have managed to achieve a life that I am satisfied with, and I hope my experiences will help others to do the same.
